# The potential preventive effect of probiotics, prebiotics, and synbiotics on cardiovascular risk factors through modulation of gut microbiota: A review

**DOI:** 10.1002/fsn3.4142

**Published:** 2024-05-29

**Authors:** Fahimeh Ghanbari, Samira Hasani, Zahra Sadat Aghili, Sedigheh Asgary

**Affiliations:** ^1^ Applied Physiology Research Center Isfahan University of Medical Sciences Isfahan Iran; ^2^ Department of Plant and Animal Biology, Faculty of Biological Science and Technology University of Isfahan Isfahan Iran; ^3^ Department of Molecular Medicine, School of Advanced Technologies Shahrekord University of Medical Sciences Shahrekord Iran; ^4^ Isfahan Cardiovascular Research Center, Cardiovascular Research Institute Isfahan University of Medical Sciences Isfahan Iran

**Keywords:** cardiovascular disease, gut microbiota, prebiotics, probiotics, synbiotics

## Abstract

Cardiovascular diseases (CVDs) are a significant contributor to global morbidity and death, underscoring the importance of their prevention and treatment. The association between the development and progression of CVD and several risk factors has been extensively studied. Among these risk factors, the gut microbiota has garnered considerable attention of the scientific community during the last two decades. In particular, dysbiosis is directly associated with many risk factors of CVD in the host, such as diabetes. Prior research has demonstrated a robust correlation between dysbiosis and the development of CVD. Probiotics, prebiotics, and synbiotics are considered important regulators of microbiota imbalances as they increase the colonization of beneficial bacteria and thereby alter the gut microbiota. Although these beneficial effects of biotics are now widely recognized, new evidence has demonstrated that target therapy of the microbiota affects many other organs, including the heart, through a process commonly referred to as the gut–heart axis. In this review, we will discuss the potential benefits of probiotics, prebiotics, and synbiotics for the beneficial effects on cardiovascular disease by modulating gut microbiota.

## Graphical abstract

Probiotics, prebiotics, and synbiotics are considered important regulators of microbiota imbalances as they increase the colonization of beneficial bacteria and thereby alter the gut microbiota. Although these beneficial effects of biotics are now widely recognized, new evidence demonstrated that target therapy of microbiota affects many other organs, including the heart, through a process commonly referred to as the gut‐heart axis.

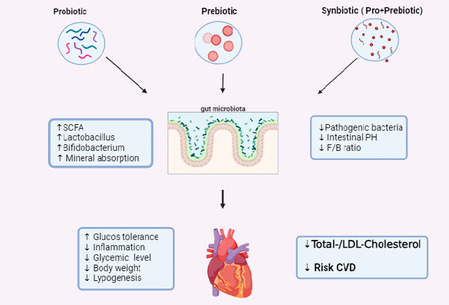

## 1 INTRODUCTION

Globally, cardiovascular diseases (CVDs) are the most prevalent noncommunicable diseases and the primary cause of mortality worldwide. The most prevalent causes of mortality were identified as acute myocardial infarction (AMI) and stroke (Agerholm‐Larsen et al., 2000; Ghorbani et al., 2023; Upadhyay, 2023). Cardiovascular diseases are often linked to different lifestyle factors, such as unhealthy dietary habits or tobacco use. These factors cause dysbiosis. These conditions are distinguished by elevated blood pressure (BP) and cholesterol levels, as well as obesity and hyperactivity of blood platelets (Tesorio et al., 2023). It is projected that the overall direct medical expenses associated with CVD will experience a threefold increase from $273 billion to $818 billion. Additionally, the actual indirect costs attributed to CVD, which are due to decreased productivity, are anticipated to rise by 61%, from $172 billion to $276 billion. The aforementioned data provide compelling evidence for the necessity of affordable and efficacious preventative and therapeutic measures for individuals afflicted with cardiovascular disease (Yoshida et al., 2018). The management of cardiovascular disease may not necessarily entail therapeutic intervention or prophylactic measures. As an illustration, it is widely acknowledged that a diet that is rich in fruits and vegetables has the potential to mitigate the likelihood of CVD through diverse pathways, such as restoring the homeostasis of the microbiota. Functional foods, commonly referred to as dietary supplements, have the potential to confer advantageous outcomes against diverse risk factors linked to cardiovascular ailments (Pimenta et al., 2018; Upadhyay, 2023). The extent to which probiotic, prebiotic, and synbiotic supplements can serve as essential dietary components in the prevention and management of CVD has not been thoroughly investigated. Several studies have demonstrated the protective effects of these interventions against CVD through mechanisms, such as cholesterol reduction, mitigation of oxidative stress, modulation of functional and structural alterations in the gut microbiota, and enhancement of immunological responses (Al Bander et al., 2020; Oniszczuk et al., 2021; Ooi & Liong, 2010). As stated by Vasquez et al. in a review article, probiotics have the potential to mitigate the production of reactive oxygen species (ROS) and alleviate oxidative stress (Vasquez et al., 2019). Despite limited research, some concise reports and minor clinical studies have been carried out, which may provide novel avenues for treatment. Furthermore, the volume of literature on these supplements, particularly with respect to the impact of biotics on cardiovascular diseases, has recently witnessed an exponential increase. The main objective of this literature review is to investigate and assess the functions and effectiveness of probiotics, prebiotics, and synbiotics in the prevention and treatment of cardiovascular disease.

## 2 PREVALENCE AND RISK FACTORS OF CVD

Cardiovascular disease (CVD) encompasses a range of pathological disorders affecting the heart and blood vessels. Coronary heart diseases, including myocardial infarction and angina, cardiomyopathy, hypertensive heart diseases, cerebrovascular diseases, deep venous thrombosis, arrhythmia, thromboembolic disease, peripheral artery diseases, rheumatic heart diseases, and pulmonary embolism, are among the most widely recognized CVDs (Hsu et al., 2021). In 2023, the World Health Organization (WHO) reported that CVDs accounted for approximately 17.9 million fatalities globally, representing approximately 32% of all deaths worldwide (Tsao et al., 2023). The etiology of the disease is contingent upon various underlying mechanisms. In general, risk factors for CVD can be categorized into two groups: nonmodifiable factors, including family history (genetic), age and gender and modifiable ones, such as hypertension, obesity, diabetes, smoking, physical inactivity, diet, cholesterol, lipids, depression, anxiety, and stress (Aggarwal et al., 2018; Balakumar et al., 2016; Simon et al., 2021). Instances, such as coronary artery disease, stroke, and peripheral artery disease, are characterized by the presence of atherosclerosis. Atherogenic indices are commonly utilized as predictors of atherosclerosis and cardiovascular diseases. These indices typically involve the ratios of total cholesterol (TC) to high‐density lipoprotein cholesterol (HDL‐C), low‐density lipoprotein cholesterol (LDL‐C) to HDL‐C, and the logarithm of triglycerides (TG) to HDL‐C (Olas, 2020; Şahin & İlgün, 2022; Tani et al., 2017). Various therapeutic options have been proposed and implemented based on risk factors associated with cardiovascular disease, with a primary emphasis on pharmaceutical interventions and modifications to one's lifestyle.

## 3 PROBIOTICS

### 3.1 History of probiotics

Probiotics, which are living microorganisms ingested for their potential health benefits, are highly regarded in the field of nutraceutical science (Hill et al., 2014). The initial introduction of the probiotic concept occurred in 1965, when Lilly and Stillwell defined probiotics as a microorganism or agents that influence the balance of the intestinal microbiota. Ilyich Metchnikoff, a Russian scientist, widely regarded as the pioneer of probiotics, published findings indicating that the abundance of *Lactobacilli* in the gastrointestinal (GI) tract has a significant impact on the well‐being of the host organism. In 1989, Fuller acknowledged the benefits of probiotics and classified them as a “viable, microbiological dietary supplement.” In 2002, the Food and Agriculture Organization (FAO)/WHO provided a definition for probiotics as “living microorganisms, such as bacteria and yeasts, that, when administered in adequate quantities and in a viable state, confer health benefits to the host” (Hill et al., 2014; Ozen & Dinleyici, 2015; Santacroce et al., 2019).

### 3.2 Characteristics of probiotics

The definition of a probiotic necessitates that it must be deemed safe for its intended application and must possess a clearly defined identity to enable accurate identification at the strain level. According to this particular definition, a probiotic is required to possess the following attributes: the optimal probiotic strains for food‐related purposes ought to originate from human sources, exhibit acid and bile resistance, have the ability to confer benefits and health advantages to the host or consumer, survive during transit through the gastrointestinal tract, adhere to the intestinal epithelial cell membrane, produce antibiotic substances to combat infections, and stabilize the intestinal microflora, and be administered at a dosage that is efficacious (Álvarez‐Arraño & Martín‐Peláez, 2021; Wu & Chiou, 2021). In order to maintain viability and prevent inactivation during transit through the gastrointestinal tract, it is imperative that the probiotics exhibit resistance to the deleterious effects of gastric juice and bile salts. Upon traversing this chemical barrier, probiotics are expected to attach to the intestinal surface, thereby enabling the manifestation of their health‐enhancing properties. Furthermore, in the event that probiotic microorganisms fail to establish a presence in the gastrointestinal tract, it may be imperative to regularly consume probiotic products. It is hypothesized that probiotics adhere to the intestinal epithelial cells, thereby impeding the proliferation of detrimental microorganisms through the production of antibacterial substances, such as ethanol, hydrogen peroxide, bacteriocins, and organic acids (Sánchez et al., 2017; Sen, 2019).

### 3.3 The mechanism of action of probiotics

According to the WHO, probiotics serve as the second most crucial immune defense mechanism when antibiotics lose their effectiveness due to antibiotic resistance. This type of treatment is commonly referred to as microbial interference therapy. Probiotics exert a favorable influence on various metabolic pathways, primarily by restoring or supporting the growth of beneficial bacteria, thereby modifying the profile of the intestinal microbiota in a health‐promoting manner (Tegegne & Kebede, 2022). Probiotic products commonly utilize seven genera, which include *Bifidobacterium, Lactobacillus, Saccharomyces, Streptococcus, Enterococcus, Escherichia*, and *Bacillus* (Kothari et al., 2019). Probiotics have been observed to enhance the nonspecific cellular immune response by stimulation of macrophages and natural killer cells, and the production of different cytokines. An increase in the number of IgA (+) cells can enhance the gut mucosal immune system (Ma et al., 2023). In addition, probiotics possess the capacity to facilitate the process of digestion and the hydrolysis of lactose, enhance the absorption of vital minerals such as manganese, calcium, iron, and zinc, and promote the biosynthesis of various vitamins such as thiamin, niacin, riboflavin, vitamin K, and pantothenic. Studies have demonstrated that probiotics possess antiproliferative, pro‐apoptotic, and anti‐oxidative characteristics (Yoha et al., 2021). Probiotics have been observed to enhance specific strains of microflora within the gastrointestinal tract, rather than the entire bacterial population (Abraham & Quigley, 2017). Certain strains exhibit distinct characteristics, such as neurological, immunological, or endocrinological effects. There may be a correlation between certain clinical advantages and the aforementioned factors. Nonetheless, it is becoming increasingly apparent that certain mechanisms of probiotic activity are common across various strains, species, and genera. The majority of *Bifidobacterium* and *Lactobacillus* strains possess the capacity to generate short‐chain fatty acids (SCFAs), including lactate and acetate, within the colon. This process is known to lower the pH of the gastrointestinal tract and is documented in the literature (Vasquez et al., 2019). SCFAs are known to enhance gut health and offer various health advantages, not only within the gastrointestinal tract but also in other areas, such as the immune system, brain, and metabolic function. Products of probiotics are predominantly accessible in the form of dietary supplements, liquids, encompassing tablets, powders, capsules, and other formulations. Commercial yogurts and cultured milk drinks, such as kefir, are fortified with probiotics (Table 1) (Hijová, 2023).

**TABLE 1:** Clinical study demonstrating effect of the probiotics, prebiotics, and synbiotics on the dysbiosis in patients.

	Type of disease	Effect of disease on microbiota	Type of biotics	Treated with biotics	References
1	Diabetes mellitus	Bifidobacterium, Faecalibacterium, Bacteroides, Akkermansia, and Roseburia ↓ Ruminococcus, Fusobacterium, and the phylum Firmicutes ↑	Probiotic	No significant differences in alpha or beta diversity of the microbiome	Sergeev et al. (2020), Zheng et al. (2018)
			Prebiotic	↑ Eubacterium eligens and Bifidobacterium	Gonai et al. (2017), Mitchell et al. (2021), Pedersen et al. (2016), Zhang et al. (2022)
			Synbiotic	↑ *Bifidobacterium* and *Lactobacillus*	Horvath et al. (2020), Kanazawa et al. (2021)
2	Hypertension	Higher (up to fivefold) Firmicutes‐to‐Bacteroides ratio Akkermansia, Bacteroides, and Clostridiaceae ↓ Streptococcus and Turicibacter ↑	Probiotic	↑ lactobacilli	Karlsson et al. (2010), Mähler et al. (2020), Robles‐Vera et al. (2020)
3	Hypercholesterolemia	Lactobacillus, Clostridium, Listeria, Bifidobacterium, and some members of Bacteroides ↓	Probiotic		NA
4	Obesity/Metabolic syndrome and lifestyle	Higher abundance of Firmicutes and lower abundance of Bacteroidetes Akkermansia muciniphila, Clostridium bartlettii, and Bifidobacterium and bacteria ↓	Probiotic	↑ Bacteroidetes ↓ Firmicutes ↓ Faecalibacterium ↓ Firmicutes/Bacteroidetes ratio	Brahe et al. (2015), Larsen et al. (2013), Leber et al. (2012), Rahayu et al. (2021), Rajkumar et al. (2014), Sun et al. (2022)
			Prebiotic	↑ SCFAs ↑ Bifidogenicity ↑ Bacteroides, Prevotella, and Porphyromonas ↓ Firmicutes ↓ Firmicutes/Bacteroidetes ratio	Everard et al. (2011), Kilic Yildirim et al. (2023), Reimer et al. (2017), She et al. (2021), Vulevic et al. (2013)
			Synbiotic	↓ fasting serum glucose levels ↓ fasting insulin levels, triglycerides, TC, and HDL cholesterol	Gutiérrez‐Repiso et al. (2019), Hibberd et al. (2019), Janczy et al. (2020), Kilic Yildirim et al. (2023)

Abbreviations: ↑, Increase; ↓, Decrease; NA, Not available; SCFAs, short‐chain fatty acids (SCFAs); TC, total cholesterol; HDL, high‐density lipoprotein.

### 3.4 Probiotic safety

As probiotics are living products, they theoretically carry the risk of long‐term effects on the microbiota, cardiometabolic, immunity, and other physiological parameters that warrant further discussion. As with most intervention studies, the long‐term safety endpoints of probiotics have been rarely tracked in studies. Recently, the issue of probiotic safety has been raised by several groups. In addition, recent clinical studies have shown significantly improved reporting of probiotic side effects (Hill, 2020; Rouanet et al., 2020; Sanders et al., 2016; van den Nieuwboer & Claassen, 2019; Žuntar et al., 2020). Concerns about probiotic safety have been raised by researchers, physicians, and policymakers. These are broadly characterized as concerns about probiotic strains, product quality, or probiotic administration. Whole genome sequencing serves as the foundation for assessing the safety of particular probiotic strains (Cohen, 2018; Freedman et al., 2020; Poindexter, 2021). The genome may also be screened for any problematic genes, such as those linked to toxicity, virulence, and antibiotic resistance (AR). One of the concerns is that genes of antibiotic resistance transferred by probiotics can be transferred to resident potential pathogens, other hostborne microbes, and/or environmental microbes, thereby transferring antimicrobial agents. A theoretical situation in which the ecosystem pool of resistance genes increases (Cohen, 2018). Another specific worry that arises is the existence of undesired living microbial contamination. Due to the fact that probiotics are intended to be provided in the form of living microorganisms, the presence of pathogenic or possibly pathogenic microorganisms presents a higher level of risk compared to products that have undergone sterilization procedures. The microbiome exerts both direct and indirect influences on medication metabolism, hence impacting both the effectiveness and potential adverse effects of drugs. The potential impact of probiotics on the functionality of drugs may have significant implications for ensuring safety (Merenstein et al., 2023). Based on short‐term observations, it has been found that specific strains of probiotics have the potential to function as opportunistic pathogens among groups characterized by advanced age, heightened stress levels, impaired immune systems, or neonatal status. The standards for testing can be customized to suit different items, with products designed for vulnerable populations undergoing more stringent testing protocols compared to those intended for the general public. The potential presence of microbial contamination in the final product, as well as the existence of allergens and other contaminants, are matters of concern. This holds true for probiotics, as well as for other therapies (Janczy et al., 2020; Ma et al., 2023). It has been hypothesized that there is a single major risk factor in vulnerable populations: for example, immunocompromised conditions, or a combination of these. Although the risk factor is low, caution should be exercised when using probiotics. Adverse events included sepsis, life‐threatening pneumonia, and endocarditis (Doern et al., 2014; Franko et al., 2013). However, probiotics may be beneficial for some vulnerable populations. Discontinuation of probiotic use should be considered unless there is compelling evidence that, based on available data, additional monitoring is required when administering probiotics to vulnerable populations (Boyle et al., 2006). Safety issues in formulating products of probiotics include the need to determine potency (the amount of live microorganisms released), composition of the final product, and purity. Additionally, probiotic products must undergo appropriate testing for potential contaminants. Furthermore, it is imperative that probiotic products undergo thorough testing to identify and assess any potential contaminants that may be present, ensuring their suitability for the intended purpose. In their study, Pariza et al. introduced a decision tree consisting of 15 levels, which serves as a valuable tool for conducting safety evaluations of items lacking a documented record of safe usage (Pariza et al., 2015).

## 4 PREBIOTICS

The notion of prebiotics is relatively more recent compared to that of probiotics. The most recent definition characterizes them as a substrate that is specifically utilized by microorganisms residing within the host, leading to beneficial effects on the overall health of the host. All molecules that are presently classified as prebiotics fall into one of the two categories: carbohydrates that can be metabolized by the gut flora, or fermentable dietary fiber (Yadav et al., 2022). The majority of prebiotics belong to the category of dietary fiber, indicating that they undergo no digestion or absorption in the small intestine; however, it is important to note that not all dietary fibers possess prebiotic properties. Prebiotics are a type of dietary fiber that is readily fermentable, providing a source of nourishment for microorganisms located in the distal bowel (Gibson et al., 2017).

These microorganisms are selectively or specifically nourished by prebiotics, which are accessible to them in the colon. Resistant starch is a variety of carbohydrates that exhibits resistance to the process of digestion in the small intestine, and instead undergoes fermentation in the large intestine. This process results in the production of prebiotic substances that serve as a source of nourishment for the advantageous *Bifidobacteria* and *Lactobacilli* microorganisms that reside within the gut. Inulin, which is naturally present in chicory root, asparagus, Jerusalem artichokes, and leeks, along with oligofructose found in bananas, wheat, garlic, honey, and onions, are among the frequently utilized carbohydrates. Additionally, galacto‐oligosaccharides (GOS), xylo‐oligosaccharides, and lactulose, a synthetic disaccharide employed as a medicinal remedy for constipation, are also noteworthy examples of commonly employed carbohydrates. Polyunsaturated fatty acids (PUFAs) and polyphenols are among the dietary compounds that could be considered as prebiotics (Yadav et al., 2022). The conversion of these chemicals into conjugated fatty acids has been demonstrated to stimulate the proliferation of advantageous bacteria in the gastrointestinal (GI) tract. The processes by which probiotics and prebiotics exert their effects are closely associated with their impact on the microbiota residing in the GI tract (Ma et al., 2023).

## 5 SYNBIOTICS

Synbiotics have become increasingly popular in recent decades due to their beneficial impact on the gut microbiota. Synbiotic formulations, which employ the amalgamation of probiotic and prebiotic, refer to a combination of live microorganisms and substrates that are specifically utilized by microorganisms residing in the host organism (Akter et al., 2021; Hijová, 2023). It is widely recognized that the administration of probiotics (live microorganisms), prebiotics (fermented ingredients), and synbiotics (a combination of pro/prebiotics) can collectively exert a modulatory effect on the gut microbiota, facilitate the proliferation of commensal microorganisms, and augment the synthesis of advantageous metabolites and as such, these interventions are frequently employed in the prevention and treatment of various diseases in pediatric populations, including infants and adolescents (Hijová, 2023). Several recent studies have suggested that they could be used as safe next‐generation treatments for a variety of diseases. The addition of pro/pre/synbiotics has the potential to address several obstacles associated with current therapies for type 2 diabetes, such as persistent adverse effects, the elevated expense of novel medications, limited patient self‐efficacy, and the requirement for lifelong medication adherence (Bock et al., 2021).

## 6 ROLE OF THE GUT MICROBIOTA IN CVD

**FIGURE 1:**
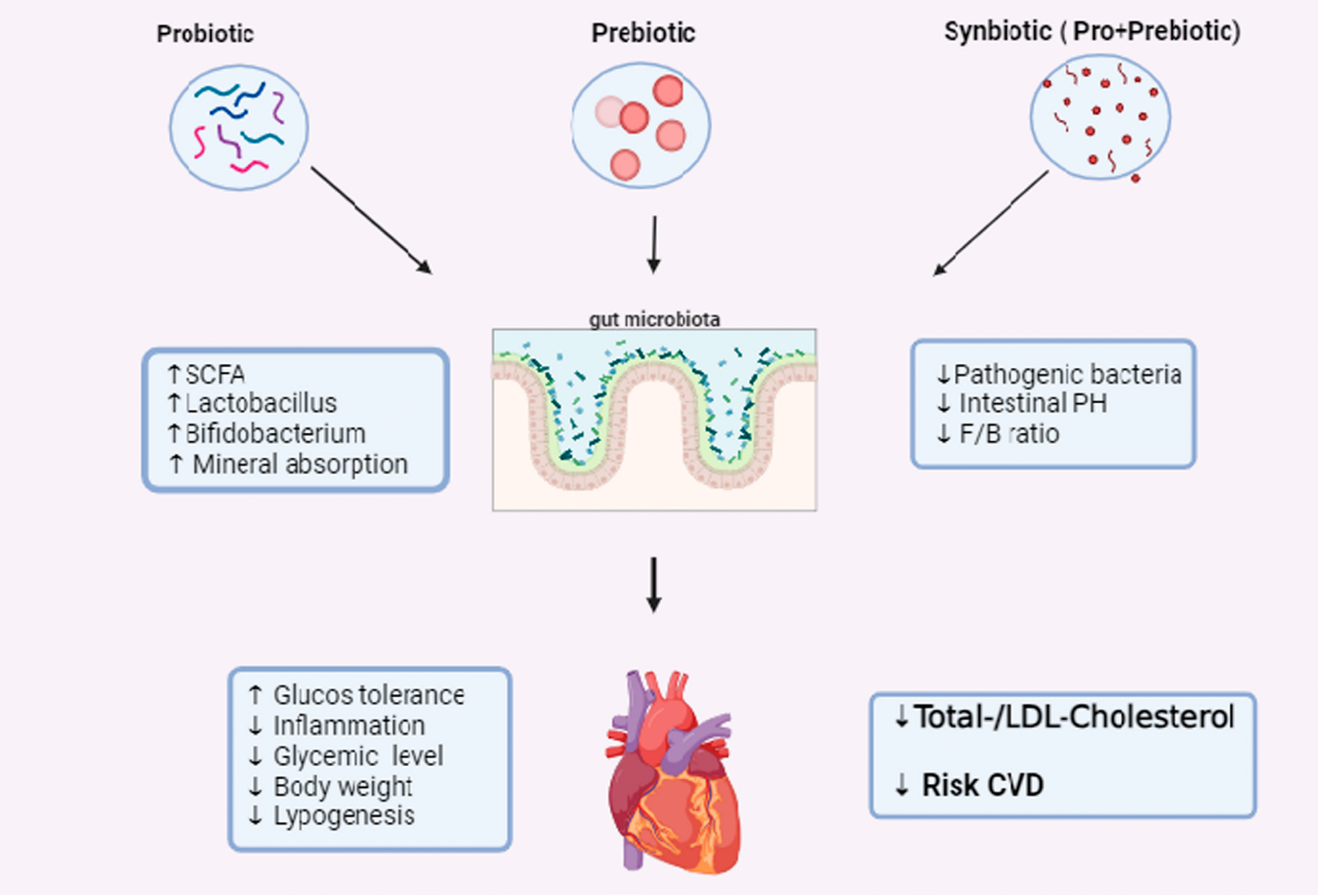
Graphical representation of effect of the probiotics, prebiotics, and synbiotics on CVD. SCFAs (short‐chain fatty acids).

The gastrointestinal tract harbors a heterogeneous population of microorganisms, comprising bacteria, viruses, fungi, and protozoa, gut microbiota, which collectively amount to approximately 100 trillion and encompass at least 1000 unique species (Singh et al., 2023; Wang et al., 2017). The interindividual variability in the gastrointestinal microbiota is remarkable, as each individual possesses a unique and distinct microbial profile. The microbial composition is observed to vary based on demographic factors such as age, gender, ethnicity, and geographic location and is influenced by factors such as genotype, initial colonization during birth, and dietary habits (Wang et al., 2017). The fecal composition of healthy adults exhibits temporal stability. Nevertheless, there exist variations in the populations of colonizing microorganisms among healthy individuals and those who suffer from diseases or poor health conditions. Notwithstanding, scholars have yet to fully delineate the constitution of a salubrious human microbiota. The human gut is predominantly inhabited by two bacterial divisions, namely *Bacteroidetes* and *Firmicutes*, which collectively constitute over 90% of the microbial population (Valdes et al., 2018). Recent research has revealed a correlation between disruptions in the gut microbiota and specific cardiovascular conditions, particularly atherosclerosis, coronary artery disease, and chronic heart failure, mediated by the cardio‐gut axis (Kumar et al., 2021; Papadopoulos et al., 2022). The gut microbiota produces metabolites that have significant implications for the prediction, improvement, and mitigation of cardiovascular disease. The intestinal flora is responsible for the production of significant metabolites, including trimethylamine N‐oxide (TMAO) and short‐chain fatty acids (SCFAs) (Yoshida et al., 2018). TMAO is a potential metabolite that is a nontraditional CVD biomarker. TMAO is associated with increased atherosclerosis, platelet hyperreactivity (via calcium release), the formation of cholesterol‐laden macrophage foam cells, vascular inflammation, and inflammasome activation (Papadopoulos et al., 2022; Zhu et al., 2016). Consequently, several large clinical cohorts have demonstrated a significant involvement of TMAO in many CVD phenotypes, including coronary artery disease (CHD), myocardial infarction and ischemic stroke, heart failure, acute coronary syndrome, and peripheral arterial disease (Haghikia et al., 2018; Jin et al., 2020; Wang et al., 2020). Changes in the ratio of *Firmicutes* to *Bacteroidetes*, indicative of intestinal dysbiosis, lead to an increase in pathogenic species and a decrease in the abundance of beneficial microbes. mainly through changes in the production of the major microbial metabolites SCFA and TMAO. Chronically affects host physiology. Therefore, a decrease in butyrate‐producing bacteria, such as *Coprococcus, Bacteroides, Butyrivibrio, Akkermansia, Lachnospiraceae*, and *Eubacterium*, is leading to various inflammatory diseases such as hypertension, obesity, and diabetes, all of which are risk factors for cardiovascular disease (Bui et al., 2023). Moreover, a disordered microbial community in the gut can directly impair cardiovascular function. Atherosis, coronary artery disease, chronic heart failure, and atrial fibrillation are diseases in which vascular inflammation due to oxidative stress and intestinal dysbiosis is common. Despite the wide range of pharmaceutical interventions available for the management of CVD, the clinical outcomes frequently fall short of being satisfactory. The incorporation of novel techniques into clinical practice has become a matter of significant urgency (Kumar et al., 2021). Probiotics demonstrate diverse mechanisms of action, primarily commencing their impacts within the gastrointestinal (GI) tract, although not limited to it. This has been documented in the literature. Biotics (probiotics, prebiotics, and synbiotics) consumption by targeting the microbiota can give promising results in preventing and treating CVD (Jenkins & Mason, 2022) (Figure 1).

## 7 CLINICAL APPLICATIONS OF PROBIOTICS AND PREBIOTICS

Probiotics have been investigated for their potential therapeutic effects in various conditions, such as antibiotic‐associated diarrhea, irritable bowel syndrome (IBS), inflammatory bowel disease (IBD) (Hempel et al., 2012; McFarland, 2010), atopic eczema in children (Boyle et al., 2009), allergies (Cuello‐Garcia et al., 2016), respiratory tract infections (Zhao et al., 2022), obesity and metabolic disease/type 2 diabetes (Homayouni et al., 2012), cardiovascular disease (Wang et al., 2018), cognitive and mental health (Ng et al., 2018), and bone health (Bonjour, 2005). Although certain systematic reviews and meta‐analyses have demonstrated favorable outcomes of probiotics in these particular conditions, the overall body of evidence is typically less robust compared to that of gastrointestinal conditions. Moreover, numerous studies have been conducted to examine the impact of probiotics, prebiotics, and synbiotics on risk factors associated with cardiovascular disease, including blood pressure, cholesterol levels, diabetes, and inflammation (Cardoso Umbelino Cavallini et al., 2016; Ebrahimi et al., 2017; Hadi et al., 2022; Raygan et al., 2018). As an illustration, a comprehensive synthesis of 13 randomized controlled trials revealed that the administration of probiotics yields a noteworthy reduction in systolic blood pressure. Notably, individuals with elevated baseline blood pressure levels exhibited a more pronounced effect (Khalesi et al., 2014). A subsequent meta‐analysis comprising 26 randomized controlled trials revealed that the administration of probiotics yields a notable reduction in both total cholesterol and LDL‐cholesterol levels, particularly among individuals with preexisting elevated cholesterol levels (Guo et al., 2011). In addition to exerting an impact on conventional cardiovascular risk factors, probiotics and prebiotics may potentially confer additional advantageous effects on the cardiovascular system. Several studies have indicated that the consumption of probiotics may have a positive impact on endothelial function, a critical indicator of cardiovascular well‐being. Endothelial dysfunction is typified by compromised vasodilation of blood vessels, resulting in elevated blood pressure and diminished coronary blood flow (Dixon et al., 2020). Metabolic syndrome is a collection of risk factors associated with CVDs, encompassing abdominal obesity, hypertension, glucose intolerance, and lipid disorders (Grundy, 2006; Rubin et al., 2022). Obesity is a prevalent health issue in contemporary nations, and it is linked to an elevated susceptibility to cardiometabolic consequences, including type 2 diabetes, hypertension, and dyslipidemia. Several systematic reviews conducted recently have indicated that the consumption of probiotics and synbiotics is associated with weight loss among overweight or obese people. However, the magnitude of weight loss, as well as the reduction in waist circumference and visceral fat, is modest (Borgeraas et al., 2018; Soltani et al., 2023; Tenorio‐Jiménez et al., 2020; Zhang et al., 2016). Research findings indicating favorable results in the treatment of obesity have predominantly employed *Lactobacillus* strains. The precise mechanisms by which probiotics elicit their advantageous effects on cardiometabolic parameters remain incompletely elucidated. However, plausible pathways encompass the modification of the gut microbiota, enhancement of intestinal barrier function, and attenuation of inflammatory cytokines. However, the lack of homogeneity in the studies included in these systematic reviews poses a challenge to establishing a clear understanding of the potential benefits of probiotics in relation to obesity and cardiometabolic parameters. The meta‐analyses indicate that the strains that exhibit the highest efficacy are *Lactobacillus acidophilus, Bifidobacterium lactis*, and *Lactobacillus plantarum* (Borgeraas et al., 2018; Soltani et al., 2023; Tenorio‐Jiménez et al., 2020; Zhang et al., 2016).

Depression and anxiety are psychological factors that have been found to have a detrimental impact on the prognosis of CVDs and are associated with an increased risk of morbidity and mortality (Roest et al., 2010). Previous research has demonstrated that the ingestion of functional foods that contain probiotics and prebiotics can have a positive impact on the mental well‐being of individuals with disrupted microbiota balance (Talbott et al., 2019). Chronic inflammation is widely recognized as a significant risk factor for CVDs and depression. Research findings have demonstrated that the utilization of probiotics has been associated with improvements in inflammatory markers, specifically C‐reactive protein, as well as enhancements in glycemic control and insulin metabolism in individuals diagnosed with metabolic syndrome or type 2 diabetes. This includes pregnant women who have been diagnosed with gestational diabetes. These findings have been reported in several studies (Guo et al., 2011; Karamali et al., 2016; Mohamadshahi et al., 2014; Sabico et al., 2019).

Additionally, dyslipidemia is acknowledged as one of the foremost risk factors associated with CVDs. Previous research has indicated that the ingestion of functional products that contain probiotic bacteria can lead to a decrease in serum lipid levels and the amelioration of dyslipidemic symptoms (Jones et al., 2012). Studies have demonstrated that probiotics have a positive effect on CVDs, by improving indicators related to CVDs, such as hypertension and dyslipidemia (Cho & Kim, 2015; Guo et al., 2011; Khalesi et al., 2014; Zhang et al., 2016). The inclusion of probiotic and synbiotic‐containing functional foods in dietary regimens has been found to have a preventive and risk‐reducing effect on CVDs. The efficacy of various probiotic bacteria in managing dyslipidemia remains uncertain. Nevertheless, certain combinations of probiotic bacteria, including *Lactobacillus reuteri NCIMB 30242, Enterococcus faecium, Lactobacillus acidophilus La5, and Bifidobacterium lactis Bb12*, have demonstrated effectiveness in mitigating dyslipidemia (Jones et al., 2012). Previous studies have indicated that the strains of *Bifidobacteria* and *Lactobacillus* have been associated with a notable reduction in serum cholesterol levels (Rubin et al., 2022). The regular consumption of kefir has been shown to have a positive impact on reducing total cholesterol and LDL‐cholesterol levels in blood lipid profiles, particularly in individuals experiencing dyslipidemic symptoms (Yilmaz & Arslan, 2022).

## 8 CONCLUSIONS

**FIGURE 2:**
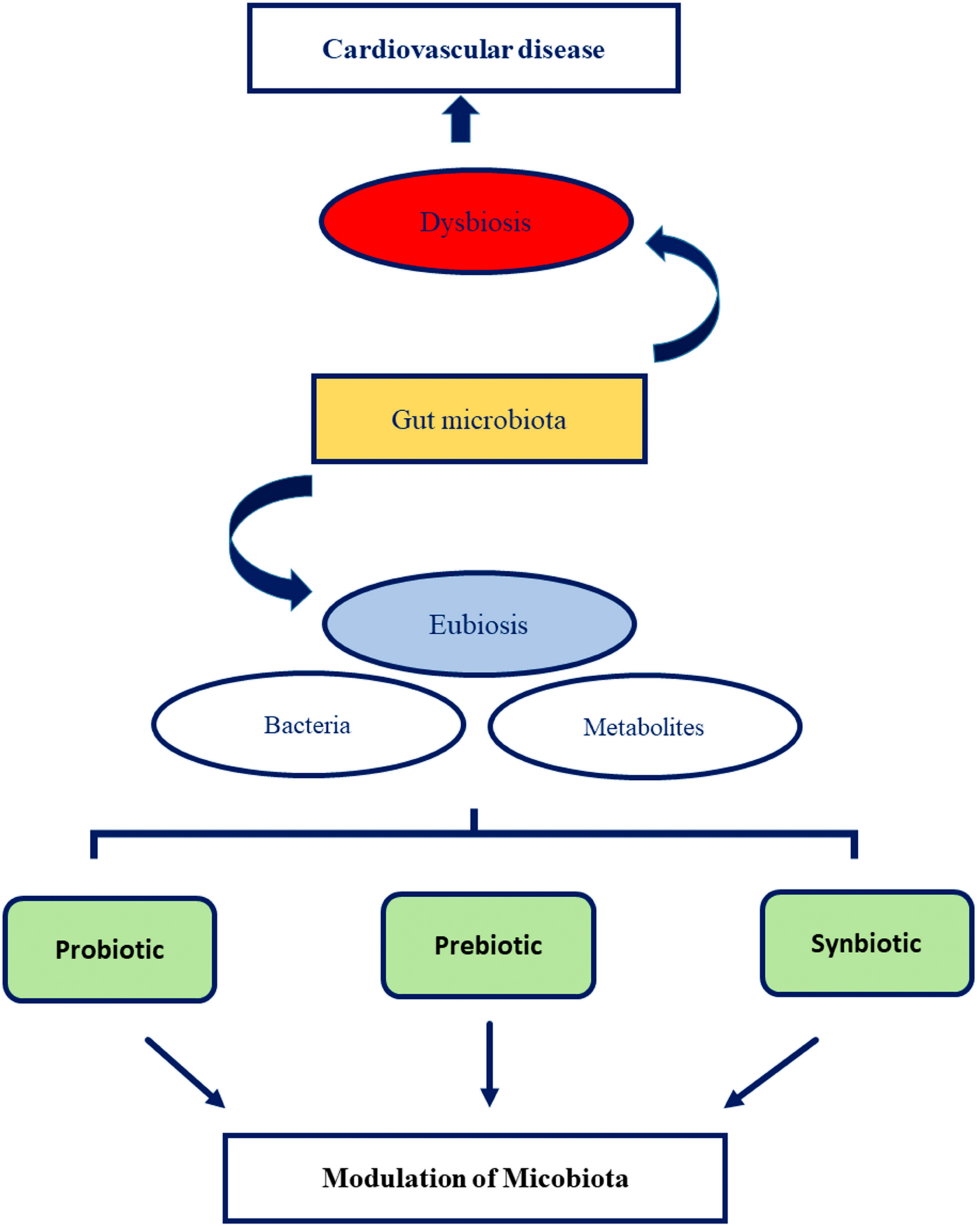
The mechanism diagram of the effect of probiotics, prebiotics, and synbiotics for the beneficial effects on cardiovascular disease by modulating gut microbiota.

The dysbiosis of gut microflora has been well recognized as an important risk factor in the pathogenesis of cardiovascular illnesses. Research has demonstrated that metabolites generated by the gut microflora play an important role in the development of cardiovascular illnesses. Consequently, the modulation of gut microflora has emerged as a promising therapeutic strategy in the treatment of these conditions. In light of prior research, it is imperative to undertake additional investigations in order to ascertain the causal, correlational, or consequential nature of the pathways between dysbiosis of the gut microbiota and cardiovascular illnesses. The utilization of agents such as prebiotics, probiotics, and postbiotics has demonstrated advantageous outcomes in the context of cardiovascular illnesses that are influenced by gut bacteria (Figure 2). In general, although the potential health advantages of probiotics and prebiotics appear promising, further investigation is required to comprehensively comprehend their underlying mechanisms and clinical implications. Furthermore, it is crucial to acknowledge that there exists variability among probiotics and prebiotics, as not all are of equal quality. The impact of these substances can differ based on several factors, including the particular strains employed, the quantity and duration of supplementation, as well as the individual's microbiota composition and overall health condition.

## AUTHOR CONTRIBUTIONS

**Fahimeh Ghanbari:** Investigation (equal); writing – original draft (equal); writing – review and editing (equal). **Samira Hasani:** Writing – original draft (equal); writing – review and editing (equal). **Zahra Sadat Aghili:** Investigation (equal); writing – review and editing (equal). **Sedigheh Asgary:** Project administration (supporting); writing – review and editing (equal).

## CONFLICT OF INTEREST STATEMENT

The authors declare that they do not have any conflict of interest.
